# Contrasting Inter- and Intraspecies Recombination Patterns in the “Harveyi Clade” *Vibrio* Collected over Large Spatial and Temporal Scales

**DOI:** 10.1093/gbe/evu269

**Published:** 2014-12-19

**Authors:** Henryk Urbanczyk, Yoshitoshi Ogura, Tetsuya Hayashi

**Affiliations:** ^1^Department of Marine Biology and Environmental Sciences, Faculty of Agriculture, University of Miyazaki, Japan; ^2^Division of Microbial Genomics, Department of Genomics and Bioenvironmental Science, Frontier Science Research Center, University of Miyazaki, Japan; ^3^Division of Microbiology, Department of Infectious Diseases, Faculty of Medicine, University of Miyazaki, Japan

**Keywords:** *Vibrio*, recombination, bacterial species definition, bacterial speciation

## Abstract

Recombination plays an important role in the divergence of bacteria, but the frequency of interspecies and intraspecies recombination events remains poorly understood. We investigated recombination events that occurred within core genomes of 35 *Vibrio* strains (family *Vibrionaceae*, Gammaproteobacteria), from six closely related species in the so-called “Harveyi clade.” The strains were selected from a collection of strains isolated in the last 90 years, from various environments worldwide. We found a close relationship between the number of interspecies recombination events within core genomes of the 35 strains and the overall genomic identity, as inferred from calculations of the average nucleotide identity. The relationship between the overall nucleotide identity and the number of detected interspecies recombination events was comparable when analyzing strains isolated over 80 years apart, from different hemispheres, or from different ecologies, as well as in strains isolated from the same geographic location within a short time frame. We further applied the same method of detecting recombination events to analyze 11 strains of *Vibrio campbellii*, and identified disproportionally high number of intraspecies recombination events within the core genomes of some, but not all, strains. The high number of recombination events was detected between *V. campbellii* strains that have significant temporal (over 18 years) and geographical (over 10,000 km) differences in their origins of isolation. Results of this study reveal a remarkable stability of Harveyi clade species, and give clues about the origins and persistence of species in the clade.

## Introduction

Microbial taxonomy classifies bacteria into distinct clusters that are referred to as species. The current species definition is practical, formed based on observations made since the early days of microbiology (Drews 2000; Tindall et al. 2010). Early bacteriologists observed phenotypic similarities between some bacterial isolates, and started classifying bacteria into species based on their phenotypic characters (Drews 2000). Exhaustive analyses of phenotypic characters such as morphology, growth rates, nutrient uptake, and pathogenicity convinced microbiologists that bacteria form distinct clusters, which correspond to species observed in multicellular organisms. Microbial taxonomists later supplemented the analyses of phenotypic characters with chemotaxonomy, DNA–DNA hybridization, analysis of 16S rRNA gene sequence, and multilocus sequence analysis (MLSA). Eventually, microbial taxonomy developed polyphasic approaches which take into account all of available information to classify strains into species (Vandamme et al. 1996; Stackebrandt et al. 2002). The use of polyphasic approaches for taxonomic classification is practical, as it allows classifying strains into species based on well-defined criteria, and allows for relatively easy replication of tests used in the classification. Use of polyphasic taxonomy allowed description of numerous new bacterial species and led to changes in the taxonomy of some bacterial groups, but the core of bacterial taxonomy remained relatively unchanged in the last 50 years.

However, the current definition of bacterial species, and even the very idea that bacteria can be classified into species came into question with the advent of whole-genome sequencing and analysis of whole-genome sequence data (Doolittle and Bapteste 2007). Analysis of genome sequence data found evidence for widespread recombination among bacteria, including frequent recombination between bacteria classified as members of different species (Feil and Spratt 2001; Kunin et al. 2005). It was predicted that a high rate of recombination between species could lead to blurring of bacterial clusters, so-called “fuzzy” species (Hanage et al. 2005; Hanage 2013). Studies of a variety of bacteria groups recognized that the recombination rates vary greatly, and may depend on ecological and biological barriers (Fraser et al. 2009; Polz et al. 2013). Importantly, recombination may lead to changes in bacterial phenotypes (Hacker and Carniel 2001), which in turn could make the taxonomic classification of bacteria into species based on phenotypic characters unreliable. Furthermore, recombination was reported in sequences used for MLSA, and within sequences coding for 16S rRNA, which brought into question usefulness of sequence analyses in taxonomic classification (Smith et al. 1999; Gevers et al. 2005).

Some models of bacterial species attempted to incorporate recombination into concepts analogous to biological species concept developed for multicellular eukaryotes (Dykhuizen and Green 1991). It was proposed that recombination, which is predicted to be proportional to the overall level of sequence similarity between genomes (Zawadzki et al. 1995; Majewski et al. 2000), could allow maintaining genetic isolation between different species, and force cohesion among populations of bacteria (Fraser et al. 2007; Caro-Quintero et al. 2011). In this scenario, recombination would allow the formation of cohesive bacterial groups, which would be comparable to multicellular eukaryotic species. This concept of bacterial species was contrasted with the ecotype model, where a species is defined as an occupant of a specific ecological niche, where periodic selection allows formation and persistence of genetic clusters (Koeppel et al. 2008; Fraser et al. 2009; Shapiro and Polz 2014). However, it is unclear how either of those models corresponds to the species definition currently used by bacterial taxonomy.

The general significance of recombination in the formation of distinct clusters of bacteria, stability of those clusters, and the clusters relation to bacterial species defined using polyphasic taxonomy remains unclear. In order to interpret the species definition used by microbial taxonomists in the light of ongoing recombination between bacteria, it is important to analyze the frequency of recombination between clusters of strains described by microbial taxonomists as species. In this regard, marine bacteria in the genus *Vibrio* (family *Vibrionaceae*, Gammaproteobacteria) appear to be one of the best models. The genus is very diverse, with over 100 described species, ubiquitous in the marine environment, and bacteria in the genus have been found occupying various ecological niches (Thompson et al. 2004; Ceccarelli and Colwell 2014). *Vibrio* have been found as planktonic bacteria, as well as forming associations with a variety of animals (Takemura et al. 2014). Some *Vibrio* are pathogens of marine fishes, squids and corals, and the genus also includes important human pathogens (Thompson et al. 2004). Species in the genus have been divided into several complexes, including so-called “Harveyi clade” (Sawabe et al. 2013; Urbanczyk et al. 2013). Several species have been included in the Harveyi clade, with the core of the clade consisting of five closely related species, *Vibrio harveyi*, *Vibrio campbellii*, *Vibrio owensii*, *Vibrio jasicida,* and *Vibrio rotiferianus*. Importantly, the bacteria in the Harveyi clade have been studied since the early days of microbiology (Figge et al. 2011; Robertson et al. 2011), and numerous strains collected from a wide variety of locations and ecologies in the last 90 years are available for studies.

Previous studies have found a variety of recombination frequencies between *Vibrio* bacteria (Shapiro et al. 2012; Shapiro and Polz 2014). High frequency of recombination events was observed within the populations of *Vibrio* occupying the same ecological niche, with frequent exchange of niche-adaptive genes (Shapiro et al. 2012; Shapiro and Polz 2014). These recombination events were observed between bacteria classified as members of the same species, in strains isolated from a relatively limited geographic location and collected within a short time frame. These recombination events were predicted to propagate and maintain intraspecies genetic diversity, and were mainly occurring in the flexible parts of the *Vibrio* genome.

In an attempt to analyze the effect of recombination on the emergence of distinct bacterial clusters and the cluster relation to bacterial species delineated using polyphasic microbial taxonomy methods, we attempted to analyze the frequency of recombination events between six *Vibrio* species in the Harveyi clade. We aimed to identify genomic regions conserved in the six species, predict evolutionary relationship between the analyzed bacteria, compare the predicted phylogeny to classification using microbial taxonomy methods, and identify the interspecies recombination events occurring within core genomes of the Harveyi clade. The same method of detecting recombination was also applied to identify intraspecies recombination events within one of the six *Vibrio* species. These analyses focused on bacteria isolated worldwide, from various habitats, and over 80 years apart.

## Materials and Methods

### Strains

A collection of 15 *Vibrio* strains was obtained from various sources (see table 1). Nine strains were isolated from seawater samples collected off the coast of Miyazaki (Japan). The seawater samples were collected along 9 km of coastline, within 25-month period. Isolation of bacteria from seawater was done using the method described by Dunlap and Urbanczyk (2013). Six other strains were obtained from different collections (table 1). All 15 strains were grown at 24–28 °C on the LSW-70 broth, as described previously (Dunlap and Urbanczyk 2013). In addition to the 15 strains, genome sequence data for additional 20 *Vibrio* strains from the Harveyi clade were obtained from public databases. Together, the 35 strains represent a worldwide collection of *Vibrio* genome sequences, isolated from various environments within the last 90 years.

**Table 1:** Bacterial Strains Analyzed in This Study

Species	Strain	Year of Isolation	Ecological Information	Accession Number(s)	Reference
*Vibrio campbellii*	NBRC 15631^T^	Before 1971	Directly from seawater, Pacific	BAOF00000000	Baumann et al. 1971
*V. campbellii*^a^	051011E	2011	Directly from seawater, Miyazaki, Japan	BBKU01000001–BBKU01000219	This study
*V. campbellii*^a^	051011F	2011	Directly from seawater, Miyazaki, Japan	BBKV01000001–BBKV01000241	This study
*V. campbellii*^a^	051011G	2011	Directly from seawater, Miyazaki, Japan	BBKG01000001–BBKG01000209	This study
*V. campbellii*^a^	151112C	2012	Directly from seawater, Miyazaki, Japan	BBKW01000001–BBKW01000251	This study
*V. campbellii*	200612B	2012	Directly from seawater, Miyazaki, Japan	BANY00000000	Urbanczyk et al. 2013
*V. campbellii*	ATCC BAA-1116	1993	Ocean isolate, USA	NC_022271, NC_022270, NC_022269	Bassler et al. 1993
*V. campbellii*^a^	CCS02	2007	Diseased fish skin (*Lates calcarifer*), Australia	BBKX01000001–BBKX01000182	Cano-Gomez et al. 2011
*V. campbellii*	DS40M4	Before 2010	Directly from seawater, Africa	AGIE00000000	Sandy et al. 2010
*V. campbellii*	HY01	2004	Dead, luminescing shrimp isolate, Thailand	AAWP00000000	Lin et al. 2010
*V. campbellii*	PEL22A	Before 2012	Directly from seawater, Brazil	AHYY00000000	Amaral et al. 2012
*V. harveyi*	NBRC 15634^T^	1935	Dead, luminescing amphipod *(Talorchestia* sp.), USA	BAOD00000000	Johnson and Shunk 1936
*V. harveyi*^a^	823TEZ1	2005	Gill of moribound abalone (*Haliotis discus hannai*), Japan	BBKY01000001–BBKY01000337	Thompson et al. 2007
*V. harveyi*	AOD131	Unknown	Diseased giant grouper (*Epinephelus lanceolatus*), Taiwan	AOMR00000000	
*V. harveyi*	CAIM 1792	2005	Diseased shrimp (*Litopenaeus vannamei*), Mexico	AHHQ00000000	Lin et al. 2010
*V. harveyi*	VHJR7	2008	Diseased *Lates calcarifer*, Malaysia	CAUO00000000	Ransangan et al. 2012
*V. harveyi*	ZJ0603	2008	Diseased orange-spotted grouper (*Epinephelus coioides*), China	AKIH00000000	Huang et al. 2012
*V. jasicida*	LMG 25398^T^	1999	Healthy packhorse lobster (*Jasus verreauxi*), New Zealand	BAOG00000000	Yoshizawa et al. 2012
*V. jasicida*	090810c	2010	Directly from seawater, Miyazaki, Japan	BAOC00000000	Urbanczyk et al. 2013
*V. jasicida*^a^	200612G	2012	Directly from seawater, Miyazaki, Japan	BBKZ01000001–BBKZ01000079	This study
*V. jasicida*^a^	201212A	2012	Directly from seawater, Miyazaki, Japan	BBLA01000001–BBLA01000115	This study
*V. jasicida*	MWB 21	Before 1924	Directly from seawater, Netherlands	BAOA00000000	Figge et al. 2011
*V. owensii*	LMG 25443^T^	Before 2010	Diseased larvae of the ornate spiny lobster (*Panulirus ornatus*), Australia	BAOH00000000	Cano-Gómez et al. 2010
*V. owensii*^a^	051011B	2011	Directly from seawater, Miyazaki, Japan	BBLB01000001–BBLB01000133	This study
*V. owensii*	1DA3	2007	Isolated from coral (*Phyllogorgia dilatata*), Brazil	ACZC00000000	Thompson et al. 2009
*V. owensii*^a^	47666-1	Before 2010	Diseased *Penaeus monodon* larvae, Australia	BBKN01000001–BBKN01000233	Cano-Gómez et al. 2010
*V. owensii*	ATCC 25919	Before 1971	Seawater enriched with glycerol and nitrate, USA	BANZ00000000	Baumann et al. 1971
*V. owensii*	LMG 25430	2005	Isolated from healthy coral (*Mussismilia hispida)*, Brazil	BAOE00000000	Chimetto et al. 2011
*V. owensii*^a^	OCN002	Before 2012	Diseased coral (*Montipora capitata*), USA	BBKO01000001–BBKO01000198	Ushijima et al. 2012
*V. rotiferianus*	LMG 21460^T^	1999	Isolated from rotifer (*Brachionus plicatilis*), Belgium	BAOI00000000	Gomez-Gil et al. 2003
*V. rotiferianus*	DAT722	Before 2006	Aquaculture tank, Australia	AFAJ00000000	Roy Chowdhury et al. 2011
*V. rotiferianus*^a^	Oz08	2006	Moribound lobster larvae (*Panulirus ornatus*), Australia	BBLC01000001–BBLC01000964	Cano-Gomez et al. 2011
*Vibrio* sp.^a^	090810a	2010	Directly from seawater, Miyazaki, Japan	BBLD01000001–BBLD01000071	This study
*Vibrio* sp.^a^	100512A	2012	Directly from seawater, Miyazaki, Japan	BBLE01000001–BBLE01000081	This study
*Vibrio* sp.^a^	151112A	2012	Directly from seawater, Miyazaki, Japan	BBLF01000001–BBLF01000214	This study

Note.—“Years of isolation” and “Ecological information” for some strains are only an approximation based on the information available.

^a^Draft genome sequences of these strains were obtained in this study.

### Whole-Genome Sequencing

Genomic DNA was isolated using the DNeasy Blood and Tissue kit (Qiagen) according to the manufacturer’s instructions for Gram-negative bacteria. Draft genome sequences were obtained using the MiSeq platform (Illumina). Libraries preparation, sequencing, and assembly were done as described previously (Urbanczyk et al. 2013). The draft genome sequences were not annotated. The sequences were submitted to the DNA Data Bank of Japan (DDBJ), see table 1 for the accession numbers.

### DNA Sequence Analysis

Average nucleotide identity (ANI) was calculated using whole-genome sequence data for the 35 *Vibrio* strains using the JSpecies program version 1.2.1 (Richter and Rosselló-Móra 2009) with default settings for calculation of ANIb.

Sequences shared by all analyzed bacterial strains were identified by a following procedure. The BLAST 2.2.28 + package was used to create a database containing whole-genome sequences of 35 *Vibrio* strains. The nucleotide sequences of 5,428 protein-coding genes of *V. campbellii* ATCC BAA-1116 were obtained from GenBank. Coding sequences of at least 500 bp long (3,776 sequences) were used as queries in searches of the genomic database of 35 *Vibrio* strains using the BLASTN algorithm, with 80% nucleotide sequence identity cutoff. Based on the search results, 899 nucleotide sequences that had a single match in all analyzed *Vibrio* strains were selected for further analysis. The 899 nucleotide sequences were manually aligned by inferred amino acid sequences using Mesquite 2.75 (http://mesquiteproject.org, last accessed July 24, 2014). During the alignment two sequences were removed from further analyses, as matching sequences recovered by BLASTN were significantly shorter for some strains. Remaining 897 alignments were concatenated, for a total of 1,032,399 characters. It should be noted that in some alignments a small number of insertions or deletions were noted. In most cases, those insertions and deletions were only present in 1 out of the 35 strains, and were likely a result of sequencing inaccuracies. As these inaccuracies were only present in one strain they were not considered as informative during phylogenetic analyses, and did not interfere with identification of recombination events.

SplitsTree version 4.12.8 (Huson and Bryant 2006) was used to construct a NeighborNet phylogenetic network using the concatenated alignment of sequences conserved in the 35 *Vibrio* strains, using default parameters. Phi test for recombination was performed in SplitsTree using default parameters. Phylogenetic analysis using parsimony criterion was performed using TNT version 1.1 (Goloboff et al. 2008). The analysis was carried out using 100 replicates of new technology searches, followed by 10,000 replicates of parsimony ratchet. Jackknife resampling values were calculated based on 10,000 replicates with 34% chance of character deletion. Tree predicted by the phylogenetic analysis was visualized using FigTree version 1.4.0 (http://tree.bio.ed.ac.uk/software/, last accessed October 16, 2014).

Program Structure version 2.3.4 (Falush et al. 2003) was used to examine genetic structure of the Harveyi clade. Data for the analysis were prepared based on concatenated alignment of 897 conserved sequences using the xmfa2struct program (http://www.xavierdidelot.xtreemhost.com/clonalframe.htm, last accessed July 24, 2014) using default parameters. Program Structure was run using an admixture model with 20,000 burn-in period, followed by 40,000 updates. Other parameters were set to default. For concatenated alignment of sequences of 35 strains, estimation of *K* (the number of populations) was set between 4 and 8. For analysis of genetic structure of *V. campbellii*, or genetic structure of *Vibrio* sp. and *V. jasicida, K* was set between 1 and 3.

### Identification of Recombination Events

Individual alignments of 897 sequences conserved in the 35 *Vibrio* strains were used to identify recent recombination events using a modification of embedded quartet decomposition analysis, as implemented by Luo et al. (2011). The analysis was applied to two pairs of strains at the same time, each pair from a different clade. PAUP* version 4.0b10 (Swofford 2003) was used to make 1,000 bootstrap replicates of each alignment, with the cut-off value of 80%. The alignments that produced phylogeny incongruent with that predicted based on concatenated alignment were selected as representing a single recombination event. No assumptions were made about direction of recombination events.

Sequence data of 897 conserved protein-coding genes conserved in the Harveyi clade were also analyzed using ClonalFrame 1.1 (Didelot and Falush 2007). ClonalFrame runs consisted of 20,000 iterations, the first half was discarded as burn-in. Genealogies predicted by ClonalFrame were visualized using FigTree version 1.4.0 (http://tree.bio.ed.ac.uk/software/, last accessed October 16, 2014). Rate of recombination to mutation (r/m) was calculated based on ClonalFrame output as described by Ellegaard et al. (2013).

## Results

### Genome Sequencing and ANI

For the purpose of this research, we obtained draft genome sequences of 15 *Vibrio* strains from the Harveyi clade (table 1). Nine strains were isolated from seawater samples collected from the coast of Miyazaki prefecture in southern Japan. Remaining six strains were collected worldwide, using various isolation techniques (table 1). Whole-genome sequence data of additional 20 *Vibrio* strains from the Harveyi clade were obtained from public databases. The genome sequence data included the whole-genome sequences of five type strains of previously described *Vibrio* species, that is, *V. campbellii*, *V. harveyi*, *V. jasicida*, *V. owensii,* and *V. rotiferianus*. Information about origin of isolation of the 35 strains can be found in table 1.

Thirty-five *Vibrio* strains were initially classified into species based on the ANI between their whole-genome sequences. Calculations of ANI were conducted between all analyzed strains (supplementary table S1, Supplementary Material online). Based on the ANI calculations, the 35 strains could be divided into six groups, which contained strains with the ANI of 95% or higher. Five of the six groups contained a single type strain from previously described *Vibrio* species. Strains from these five groups were therefore taxonomically classified into species based on the classification of the corresponding type strains. Three strains (090810a, 100512A, and 151112A) formed a group that could not be assigned to any previously described *Vibrio* species, and will therefore be referred to as *Vibrio* sp. in this work.

### Phylogenetic Analyses

**Fig. 1.—:**
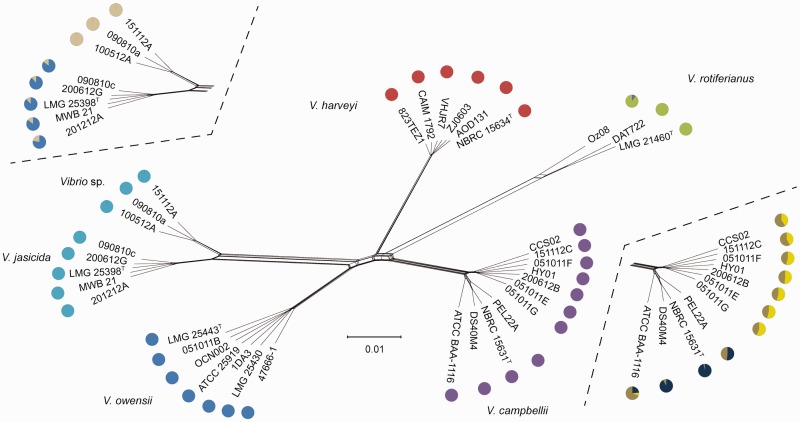
Analysis of genetic structure in the “Harveyi clade.” Outputs from Structure analysis of 35 *Vibrio* strains (represented by pie charts with colors indicating proportion of estimated ancestries from five hypothetical populations; each color represents a different ancestral population) were mapped on a phylogenetic network predicted using NeighborNet analysis. The network was constructed based on analysis of concatenated alignment of 897 protein-coding sequences conserved in the six *Vibrio* species. The scale bar shows sequence divergence. Upper inset shows the results of an additional Structure analysis that included three *Vibrio* sp. and five *V. jasicida* strains. Lower inset shows results of a third Structure analysis, which focused on 11 *V. campbellii* strains.

Nucleotide sequences of 3,776 protein-coding genes found in *V. campbellii* ATCC BAA-1116 were used as queries for blast searches of the in-house database containing the whole-genome sequence data of 35 strains from the Harveyi clade (see Materials and Methods). These searches identified 897 protein-coding sequences conserved in all 35 *Vibrio* strains. The sequences were manually aligned and the alignments were concatenated. The resulting concatenated alignment (with 1,032,399 characters) was used for NeighborNet analysis to illustrate evolutionary relationship between the analyzed strains (fig. 1). This analysis assigned the 35 strains into six clades, which corresponded to grouping of strains based on ANI. Using the same concatenated data set for 897 protein-coding sequences, a parsimonious analysis showed same assignment of strains into clades (supplementary fig. S1, Supplementary Material online). In both NeighborNet and parsimony analyses, *V. campbellii* strains were divided into two subclades. Nine *V. campbellii* strains were assigned to the same subclades in both analyses. However, assignment of strains CCS02 and 151112C to subclades was incongruent between both analyses.

### Genetic Structure of the Harveyi Clade

Program Structure was chosen to analyze the genetic structure of Harveyi clade. The analysis revealed the pattern of admixture resulting from recombination events within the 35 strains. For the analysis, the concatenated alignment of 897 conserved sequences was used. The highest likelihood was observed for *K* (the number of populations) equal 5. All strains of *V. rotiferianus*, *V. harveyi*, *V. owensii* and *V. campbellii* had well-defined ancestries, whereas *V. jasicida* and *Vibrio* sp. strains shared ancestry (fig. 1). A second Structure analysis which analyzed five *V. jasicida* and three *Vibrio* sp. strains found *K* = 2 as the best representation of ancestry of the two species, with limited mixed ancestry between the two species (fig. 1). A Structure analysis which included 11 *V. campbellii* strains revealed *K* = 3 has the highest likelihood of the data, with mixed ancestry between the 11 strains (fig. 1).

### Recombination Frequency

Phi test performed in the SplitsTree program using concatenated alignment of 897 conserved sequences found statistically significant evidence for recombination (*P* = 0.0). In order to identify interspecies recombination events between six *Vibrio* species, a method based on a phylogenetic analysis was used (see Materials and Methods). Briefly, the analysis identified recombination events between two pairs of strains, each pair of strains selected from different clades identified by the NeighborNet analysis (fig. 1). During the analysis, the assumption was that unless a recombination event occurred, results of a phylogenetic analysis of sequences of any of the 897 conserved sequences in any four strains should be congruent with phylogeny elucidated based on analysis of concatenated alignment. To test that assumption, alignments of sequences of individual genes from two pairs of strains from two different clades were bootstrapped and compared with the topology of the tree based on concatenated alignment. Results of analyses of individual genes sequences that were incongruent with those of concatenated alignment were considered to be an evidence for a recombination event between strains from the two clades. For each identified incongruence a single recombination event was assumed, with no assumption regarding direction of recombination event.

**Fig. 2.—:**
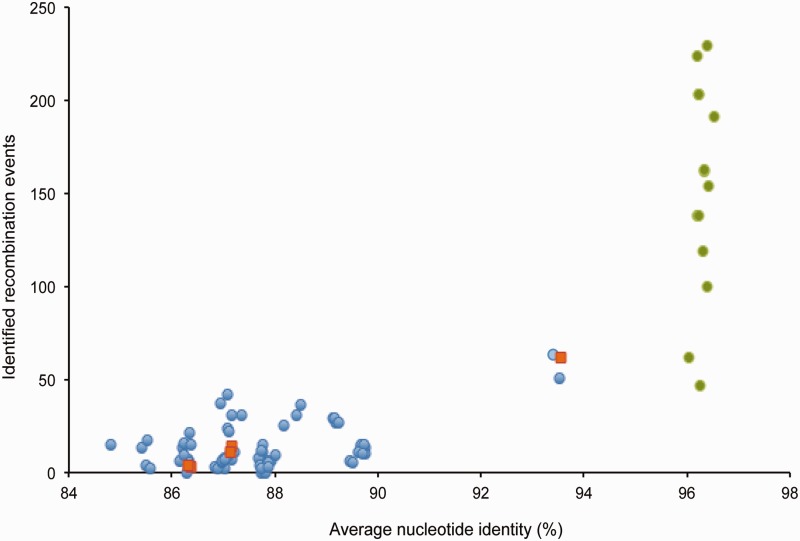
Relationship between the number of identified recombination events and the ANI. Each marker represents the number of recombination events identified between two pairs of strains from different clades plotted against average ANI between analyzed strains from different clades. Blue circles represent results of analysis of interspecies recombination events; orange squares represent results of analysis of strains isolated in the Miyazaki prefecture within a short time frame; green circles indicate results of analysis of intraspecies recombination events.

For the first analysis, pairs of strains were selected to be representative for their species. Higher priorities were given to pairs of strains isolated at distant geographical locations, from different ecological sources, using various isolation techniques, and with significant temporal differences in their origin of isolation. Information about origin of isolation of each strain is listed in table 1. The pairs of strains selected were *V. campbellii* 051011E and 200612B, *V. campbellii* 151112C and HY01, *V. campbellii* DS40M4 and NBRC 15631^T^, *V. campbellii* ATCC BAA-1116 and PEL22A, *V. campbellii* ATCC BAA-1116 and DS40M4, *V. harveyi* NBRC 15634^T^ and ZJ0603, *V. harveyi* CAIM 1792 and VHJR7, *V. jasicida* LMG 25398^T^ and 200612G, *V. jasicida* 090810C and MWB21, *V. owensii* ATCC 25919 and 051011B, *V. owensii* 1DA3 and LMG 25443^T^, *V. rotiferianus* LMG 21460^T^ and Oz08, *Vibrio* sp. 151112A and 090810a. The number of identified recombination events was plotted versus the average ANI between strains from different clades used in the analyses (fig. 2). The results revealed close relationship between the number of interspecies recombination and the overall genome sequence identity (*R*^2 ^= 0.37681, supplementary fig. S2, Supplementary Material online). Numbers of detected recombination events between each pair of strains are also listed in the supplementary table S2, Supplementary Material online.

A second analysis of recombination events included pairs of strains isolated recently (within 25 months) from the same geographical location (shallow coastal water in the Miyazaki prefecture), using the same collection technique (see Materials and Methods). The pairs of strains were *V. campbellii* 151112C and 051011E, *V. campbellii* 051011E and 200612B, *V. jasicida* 090810c and 200612G, and *Vibrio* sp. 151112A and 090810a. Strains *Vibrio* sp. 151112A and *V. campbellii* 151112C were isolated from the same 50 ml seawater sample. Similar to results of analysis of interspecies recombination in strains collected worldwide from various ecologies, analysis of strains collected in Miyazaki prefecture (fig. 2, supplementary fig. S2 and table S2, Supplementary Material online) found close relationship between the number of recombination events and overall genome sequence identity.

A third analysis focused on identification of recombination events within a single species, *V. campbellii*. The same method used to analyze interspecies recombination events between six *Vibrio* species was used to identify the number of intraspecies recombination events, in particular recombination events between *V. campbellii* strains from two different subclades. For the analysis, *V. campbellii* strains were divided into two groups, corresponding to two subclades identified using NeighborNet analysis, and the number of recombination events between strains from two different subclades was elucidated. Results of the analysis found disproportionally high numbers of intraspecies recombination events (fig. 2 and supplementary table S2, Supplementary Material online).

The disproportionally high numbers of intraspecies recombination events within *V. campbellii* were identified in the analyses that include two strains, namely ATCC BAA-1116 and PEL22A (supplementary table S2, Supplementary Material online). The same two strains have shown atypical admixture patter in the analysis of *V. campbellii* genetic structure (fig. 1, lower inset). In order to determine whether these results are due to a major recombination event involving many genes, or due to multiple small events, conserved sequences that shown evidence of recombination were mapped on strain ATCC BAA-1116 chromosomes (supplementary fig. S3, Supplementary Material online). No conserved protein-coding genes were found on the strain plasmid. Results of the mapping have found sequences showing evidence of recombination to be spread along both chromosomes of the strain ATCC BAA-1116 (supplementary fig. S3, Supplementary Material online), which suggests that the strain was involved in numerous intraspecies recombination events, and pattern of admixture in *V. campbellii* revealed by Structure analysis is not a result of a single recombination event involving large number of sequences.

### ClonalFrame Analysis

Analysis of interspecies and intraspecies recombination events in the Harveyi clade was also conducted using ClonalFrame software (Didelot and Falush 2007). ClonalFrame uses multilocus sequence data to predict clonal relationship between bacterial strains and estimates probability that a given site is altered through recombination. ClonalFrame was applied to three data sets, each containing aligned 897 protein-coding sequences conserved in the Harveyi clade. The first data set contained 11 *V. campbellii* strains, second data set included five *V. jasicida* and three *Vibrio* sp. strains, and the third data set contained three *Vibrio* sp. strains and *V. jasicida* 090810c. For each data set, genealogies predicted by the ClonalFrame software were similar to those predicted by phylogenetic and NeighborNet analyses (supplementary fig. S4, Supplementary Material online). Analyses of the first two data sets, which included multiple strains from a single species, identified high number of recombining sites in each strain, between 5,883 and 10,788 (supplementary fig. S4, Supplementary Material online). In the third analysis, which included three *Vibrio* sp. and a single *V. jasicida* strains, between 7,995 and 10,055 recombining sites were identified among *Vibrio* sp. strains, but only six recombining sites were identified in *V. jasicida* 090810c. These results can be interpreted as evidence that intraspecies recombination events have greater effect on genealogies of Harveyi clade bacteria than interspecies recombination events.

## Discussion

Reported here are the results of a series of analyses of recombination events that occurred between 35 strains representing six closely related *Vibrio* species in the Harveyi clade. The analyses focused on recombination events that occurred within core regions of the bacteria genomes. The number of identified interspecies recombination events was in all cases correlated with the overall genomic identity between the analyzed strains, with increased number of recombination events occurring between genomes with higher average nucleotide sequence identity (fig. 2). In contrast, the number of identified intraspecies recombination events between some (but not all) strains of *V. campbellii* from different subclades was disproportionally high.

In this study, the number of identified interspecies recombination events was relatively low, and related to the overall genome sequence identity (supplementary fig. S2, Supplementary Material online). ClonalFrame analysis also predicted low effect of interspecies recombination on genealogies of the Harveyi clade bacteria (supplementary fig. S4, Supplementary Material online). Furthermore, the number of identified interspecies recombination events was independent of the origin of isolation. We found a good correlation between the number of interspecies recombination events and the overall genome identity when analyzing strains isolated over 80 years apart, as well as in strains isolated only few months apart. We also found no correlation between the number of interspecies recombination events and the strains habitats, as we found comparable number of interspecies recombination events between planktonic strains and in strains associated with marine animals. We also found comparable numbers of interspecies recombination events between strains isolated from different hemispheres, or between strains isolated from a limited geographical location (i.e., 9-km coastline in the Miyazaki prefecture). A low number of interspecies recombination events was found even when analyzing strains from different species isolated from the same 50 ml sample of seawater. Despite similar ecologies and presumably ample opportunities for exchange of genetic material, the number of interspecies recombination events within core genome of the strains isolated in the Miyazaki prefecture was in all cases correlated with the overall genomes sequence identity. Many factors likely influence recombination between Harveyi clade species, but the results reported here suggest that shared ecology of bacteria had little influence over the frequency of interspecies recombination in the core genomic regions.

In this regard, it should be stressed the method used in here allows calculating frequency of recombination between sequences conserved in the six *Vibrio* species, and are a part of the bacteria core genomes. The analyzed conserved sequences had 1.03 million nucleotides, which constitutes less than 25% of most genomes used in this study. The frequency of recombination between flexible parts of the *Vibrio* genomes is likely different from that reported here. Previous studies found frequent exchange of niche-adaptive genes located in flexible parts of *Vibrio* genomes, with higher rate of recombination between strains isolated from the same ecological niche (Shapiro et al. 2012; Polz et al. 2013). Here, we found no obvious correlation between the number of recombination events in the core genomes and the bacteria ecology.

In contrast to the results of analysis of interspecies recombination events, we found a high number of intraspecies recombination events that occurred within the core genomes of some *V. campbellii* strains from two different subclades. The number of identified intraspecies recombination events was higher than would be expected based on the overall sequence identity between the strains. ClonalFrame analysis also identified high number of recombining sites in the analyses that included multiple strains from a single species. High frequency of intraspecies recombination was found between strains isolated over 18 years apart, and from distinct geographical locations. It should be noted that the disproportionally high frequency of recombination events was only observed between some, but not all *V. campbellii* strains, namely ATCC BAA-1116 and PEL22A. The two strains also show a distinct admixture pattern in the analysis of *V. campbellii* genetic structure (fig. 1, lower inset). At this moment, it is not clear why frequency of intraspecies recombination events differs between some *V. campbellii* strains. Based on the results of mapping of recombining sequences on strain ATCC BAA-1116 chromosomes, it is likely that the pattern of interspecies recombination observed in this *V. campbellii* is a result of numerous independent events, and not due to a single event involving multiple genes. Furthermore, our analysis only identified recombination events occurring between strains from different *V. campbellii* subclades, and did not estimate frequency of recombination events between strains from the same subclade. The method used in this study to identify recombination events relies on understanding of evolutionary relationship between the analyzed strains, but we were unable to confidently determine the relationship between strains from the same *V. campbellii* subclade. It is likely that the number of intraspecies recombination events between strains from the same subclade is higher than that identified between strains from different subclades.

The number of inter- and intraspecies recombination events reported here encouraged us to analyze the genetic structure of Harveyi clade using program Structure. Initially, the analysis found five ancestral populations with very limited admixture. The five ancestral populations corresponded to five out of six species identified based on results of ANI, reticulation in the NeighborNet network, and results of a parsimonious analysis. Initial Structure analysis could not distinguish two closely related species, *V. jasicida* and *Vibrio* sp. However, a closer inspection of results of Structure analysis suggested limited admixture of *V. jasicida* and *Vibrio* sp. An analysis of genetic structure that focused on *V. jasicida* and *Vibrio* sp. strains identified two ancestral populations, with only limited admixture. In contrast to the situation found in *V. jasicida* and *Vibrio* sp., analysis of a genetic structure within *V. campbellii* strains identified three ancestral populations, with high degree of admixture. Overall, we interpret results of these Structure analyses as an evidence of relatively low levels of admixture between the six *Vibrio* species, and consider the initial prediction of shared ancestry between *V. jasicida* and *Vibrio* sp. as a result of too little data available to find a true genetic structure of the 35 strains. We predict that separation of *Vibrio* sp. and *V. jasicida* was a relatively recent event, whereas separation of other species occurred earlier. Results of these Structure analyses therefore support classification of the 35 strains into six species, and are in agreement with our conclusion about the frequency of interspecies and intraspecies recombination events.

The results of this study revealed clear-cut boundaries between the analyzed *Vibrio* species. Based on the ANI (supplementary table S1, Supplementary Material online) we were able to assign all analyzed strains into six groups, which correspond to five *Vibrio* species with validly published names and one previously undescribed species. This classification was also supported by reticulation revealed by the NeighborNet (fig. 1), and the evolutionary relationship evident from the results of parsimonious analysis (supplementary fig. S2, Supplementary Material online), as well as genetic structure of the 35 strains. Relatively, low levels of identified interspecies recombination events and high frequency of intraspecies recombination events also supported classification of the 35 strains into six species. Importantly, the use of large number of sequence data for MLSA allowed good resolution of relationship between the six species, despite evidence of recombination within some of the sequences used for the analysis. Remarkably, strains analyzed in this study could be confidently classified into species despite significant geographical or temporal distance between their isolation. The strains analyzed here were sampled over 80 years apart, from distant geographic locations and from varied environments, yet the 35 strains formed well-defined, remarkably cohesive and genetically similar groups.

Results of this study revealed Harveyi clade bacteria as very stable, cohesive groups, and can be used as a basis for future studies of evolutionary processes responsible for the emergence of species in the clade. Based on results of this study, we suggest that studies of Harveyi clade diversification processes should analyze a large number of strains, isolated from a wide variety of habitats, and from diverse geographical locations. With respect to the bacterial species definition, the remarkable cohesiveness of species in the Harveyi clade was observed in groups of strains classified as members of the same species using definition currently used in bacterial taxonomy. We conclude that the current bacterial species definition adequately describes Harveyi clade diversity, and is a good starting point for discussion of speciation in other *Vibrio* clades. It remains to be determined whether other *Vibrio* species show similar stability and cohesiveness as Harveyi clade bacteria.

## Supplementary Material

Supplementary figures S1–S4 and tables S1 and S2 are available at *Genome Biology and Evolution* online (http://www.gbe.oxfordjournals.org/).

Supplementary Data
