# Supplementary material for: Contrasting Inter- and Intraspecies Recombination Patterns in the “Harveyi Clade” Vibrio Collected over Large Spatial and Temporal Scales
Source: Genome Biol Evol. 2014 Dec 19;7(1):71–80. doi: 10.1093/gbe/evu269 (PMC4316622; doi:10.1093/gbe/evu269)
Supplement: Supplementary Data [file supp_7_1_71__index.html]

Contrasting inter- and intraspecies recombination patterns in the ‘Harveyi clade’ Vibrio collected over large spatial and temporal scales. — Contrasting Inter- and Intraspecies Recombination Patterns in the “Harveyi Clade” Vibrio Collected over Large Spatial and Temporal Scales — Supplementary Data 

# Contrasting Inter- and Intraspecies Recombination Patterns in the “Harveyi Clade” *Vibrio* Collected over Large Spatial and Temporal Scales

## Supplementary Data

files

**Files in this Data Supplement:**

- Supplementary Data - pdf file
