# Supplementary material for: Contrasting Inter- and Intraspecies Recombination Patterns in the “Harveyi Clade” Vibrio Collected over Large Spatial and Temporal Scales
Source: Genome Biol Evol. 2014 Dec 19;7(1):71–80. doi: 10.1093/gbe/evu269 (PMC4316622; doi:10.1093/gbe/evu269)
Supplement: Supplementary Data [file supp_evu269_HarveyiRecombinationSupplementaryMaterial.pdf]

Supplementary Material for:

Contrasting inter- and intraspecies recombination patterns in the 'Harveyi clade' *Vibrio* collected over large spatial and temporal scales.

Henryk Urbanczyk, Yoshitoshi Ogura, Tetsuya Hayashi.

Supplementary table S1.

Supplementary table S1

Average nucleotide identities (ANI) between the analyzed strains.

Reference genome		Tester genome: *V. campbellii* NBRC 15631^{T}	*V. campbellii* 051011E	*V. campbellii* 051011F	*V. campbellii* 051011G	*V. campbellii* 151112C	*V. campbellii* 200612B	*V. campbellii* ATCC BAA-1116	*V. campbellii* CCS02	*V. campbellii* DS40M4	*V. campbellii* HY01	*V. campbellii* PEL22A	*V. harveyi* NBRC 15634^{T}	*V. harveyi* 823TEZ1	*V. harveyi* AOD131	*V. harveyi* CAIM 1792	*V. harveyi* VHJR7	*V. harveyi* ZJ0603
Vibrio campbellii	NBRC 15631^{T}	---	**96.08**	**96.10**	**96.11**	**96.09**	**96.04**	**96.58**	**96.05**	**97.88**	**95.84**	**96.75**	88.06	88.10	87.55	87.95	88.02	87.75
V. campbellii	051011E	**96.18**	---	**97.48**	**97.70**	**97.12**	**97.58**	**96.59**	**97.26**	**96.17**	**97.39**	**96.52**	88.14	88.21	87.37	88.10	88.19	87.84
V. campbellii	051011F	**96.20**	**97.50**	---	**97.56**	**97.36**	**97.52**	**96.51**	**97.43**	**96.13**	**97.45**	**96.51**	88.09	88.20	87.45	88.09	88.19	87.84
V. campbellii	051011G	**96.18**	**97.77**	**97.59**	---	**97.22**	**97.59**	**96.58**	**97.33**	**96.11**	**97.40**	**96.43**	88.18	88.19	87.56	88.13	88.17	87.85
V. campbellii	151112C	**96.11**	**97.13**	**97.30**	**97.15**	---	**97.16**	**96.26**	**97.25**	**96.02**	**96.98**	**96.28**	88.03	88.07	87.50	87.88	88.11	87.79
V. campbellii	200612B	**96.05**	**97.60**	**97.44**	**97.53**	**97.13**	---	**96.44**	**97.33**	**96.05**	**97.27**	**96.39**	87.97	88.01	87.51	87.89	88.07	87.86
V. campbellii	ATCC BAA-1116	**96.63**	**96.69**	**96.56**	**96.60**	**96.42**	**96.51**	---	**96.48**	**96.47**	**96.44**	**96.08**	88.11	88.13	87.14	87.98	88.08	87.60
V. campbellii	CCS02	**96.07**	**97.26**	**97.39**	**97.30**	**97.23**	**97.31**	**96.40**	---	**96.07**	**97.18**	**96.40**	88.06	88.13	87.47	88.02	88.19	87.92
V. campbellii	DS40M4	**97.94**	**96.14**	**96.17**	**96.14**	**96.17**	**96.14**	**96.63**	**96.13**	---	**95.99**	**96.81**	87.88	87.86	87.43	87.81	87.89	87.64
V. campbellii	HY01	**96.08**	**97.45**	**97.52**	**97.46**	**97.10**	**97.42**	**96.42**	**97.23**	**96.07**	---	**96.42**	87.85	87.86	87.37	87.83	87.91	87.64
V. campbellii	PEL22A	**96.82**	**96.58**	**96.63**	**96.53**	**96.48**	**96.51**	**96.46**	**96.54**	**96.75**	**96.41**	---	87.81	87.77	87.21	87.80	87.84	87.61
V. harveyi	NBRC 15634^{T}	87.84	87.92	87.88	87.93	87.89	87.91	87.89	87.87	87.79	87.65	87.64	---	**98.52**	**98.46**	**98.53**	**98.61**	**98.56**
V. harveyi	823TEZ1	88.16	88.35	88.32	88.35	88.30	88.29	88.31	88.30	88.06	88.09	87.87	**98.47**	---	**98.43**	**98.54**	**98.54**	**98.46**
V. harveyi	AOD131	87.27	87.40	87.53	87.54	87.54	87.43	87.31	87.48	87.21	87.20	87.17	**98.59**	**98.53**	---	**98.60**	**98.50**	**98.64**
V. harveyi	CAIM 1792	87.70	87.93	87.91	87.93	87.82	87.91	87.84	87.96	87.70	87.68	87.66	**98.66**	**98.62**	**98.65**	---	**98.70**	**98.72**
V. harveyi	VHJR7	87.79	88.00	88.05	88.00	88.02	88.06	87.90	88.06	87.78	87.71	87.77	**98.63**	**98.58**	**98.52**	**98.68**	---	**98.74**
V. harveyi	ZJ0603	87.45	87.85	87.83	87.80	87.79	87.83	87.53	87.89	87.47	87.50	87.39	**98.64**	**98.56**	**98.55**	**98.67**	**98.66**	---
V. jasicida	LMG 25398^{T}	86.91	87.08	87.07	87.12	87.25	87.22	87.15	87.05	86.78	86.78	86.83	86.31	86.25	86.06	86.21	86.29	86.15
V. jasicida	090810c	86.80	87.04	87.03	87.04	87.17	87.07	87.09	87.01	86.74	86.80	86.78	86.15	86.19	85.90	86.12	86.23	86.13
V. jasicida	200612G	86.68	86.96	87.02	86.86	87.11	87.04	86.95	87.00	86.58	86.72	86.73	86.07	86.14	85.87	86.13	86.21	86.05
V. jasicida	201212A	86.69	86.95	87.02	86.96	87.11	86.98	86.95	86.99	86.63	86.66	86.70	86.10	86.12	86.02	86.15	86.18	86.10
V. jasicida	MWB 21	86.89	87.12	87.08	87.14	87.18	87.12	87.15	87.07	86.75	86.93	86.84	86.24	86.21	86.04	86.17	86.25	86.17
V. owensii	LMG 25443^{T}	89.54	89.84	89.73	89.75	89.89	89.79	89.84	89.80	89.44	89.52	89.62	88.78	88.85	88.37	88.70	88.69	88.42
V. owensii	051011B	89.38	89.68	89.77	89.68	89.82	89.77	89.66	89.75	89.39	89.50	89.53	88.22	88.33	87.95	88.24	88.25	88.03
V. owensii	1DA3	89.46	89.62	89.72	89.60	89.81	89.65	89.71	89.69	89.48	89.39	89.62	88.37	88.53	88.01	88.34	88.36	88.17
V. owensii	47666-1	89.66	90.06	89.99	90.01	90.19	90.08	90.05	90.04	89.68	89.87	89.75	88.57	88.69	87.92	88.50	88.55	88.28
V. owensii	ATCC 25919	89.43	89.70	89.72	89.59	89.78	89.66	89.67	89.71	89.41	89.36	89.55	88.25	88.36	87.88	88.26	88.21	88.10
V. owensii	LMG 25430	89.59	89.74	89.71	89.70	89.87	89.81	89.76	89.68	89.54	89.42	89.63	88.33	88.48	87.98	88.31	88.32	88.18
V. owensii	OCN002	89.66	89.80	89.74	89.73	89.84	89.79	89.84	89.85	89.61	89.61	89.66	88.47	88.58	88.06	88.50	88.46	88.20
V. rotiferianus	LMG 21460^{T}	86.68	87.10	87.05	87.09	87.03	87.06	86.94	87.11	86.61	86.73	86.78	85.94	85.89	85.60	85.79	86.05	85.91
V. rotiferianus	DAT722	86.62	87.17	87.19	87.13	87.18	87.16	86.86	87.28	86.60	86.80	86.69	86.01	86.01	85.89	86.02	86.23	86.14
V. rotiferianus	Oz08	87.54	87.87	87.79	87.74	87.82	87.89	87.75	87.80	87.46	87.60	87.21	87.30	87.32	86.34	87.18	87.32	86.89
Vibrio sp.	090810a	86.26	86.26	86.24	86.20	86.42	86.28	86.38	86.26	86.13	86.10	86.23	85.60	85.68	85.27	85.60	85.61	85.49
Vibrio sp.	100512A	86.24	86.34	86.34	86.28	86.51	86.34	86.46	86.32	86.14	86.13	86.27	85.59	85.67	85.31	85.56	85.60	85.50
Vibrio sp.	151112A	86.26	86.25	86.38	86.38	86.40	86.37	86.30	86.24	86.17	86.07	86.29	85.55	85.62	85.22	85.51	85.59	85.40

ANI values are presented as percentage. ANI values of 95% or higher are marked in bold.

Supplementary table S1

Average nucleotide identities (ANI) between the analyzed strains.

Reference genome		Tester genome: *V. jasicida* LMG 25398^{T}	*V. jasicida* 090810c	*V. jasicida* 200612G	*V. jasicida* 201212A	*V. jasicida* MWB 21	*V. owensii* LMG 25443^{T}	*V. owensii* 051011B	*V. owensii* 1DA3	*V. owensii* 47666-1	*V. owensii* ATCC 25919	*V. owensii* LMG 25430	*V. owensii* OCN002	*V. rotiferianus* LMG 21460^{T}	*V. rotiferianus* DAT722	*V. rotiferianus* Oz08	*Vibrio* sp. 090810a	*Vibrio* sp. 100512A	*Vibrio* sp. 151112A
Vibrio campbellii	NBRC 15631^{T}	87.11	87.05	86.97	86.98	87.07	89.57	89.43	89.62	89.67	89.59	89.66	89.69	86.97	86.84	87.04	86.45	86.50	86.45
V. campbellii	051011E	87.26	87.32	87.11	87.04	87.37	89.88	89.74	89.91	90.13	89.89	89.94	89.92	87.29	87.49	87.32	86.28	86.36	86.35
V. campbellii	051011F	87.28	87.28	87.15	87.18	87.36	89.82	89.81	89.90	90.09	89.94	89.90	89.90	87.21	87.41	87.22	86.32	86.39	86.53
V. campbellii	051011G	87.26	87.30	87.03	87.19	87.36	89.82	89.76	89.82	90.07	89.79	89.82	89.82	87.35	87.46	87.20	86.30	86.31	86.51
V. campbellii	151112C	87.37	87.36	87.22	87.26	87.39	89.89	89.83	89.90	90.15	89.92	89.94	89.89	87.18	87.35	87.26	86.47	86.52	86.53
V. campbellii	200612B	87.32	87.23	87.23	87.12	87.21	89.71	89.79	89.71	90.00	89.72	89.87	89.74	87.11	87.22	87.13	86.37	86.42	86.44
V. campbellii	ATCC BAA-1116	87.33	87.19	87.10	87.11	87.29	89.79	89.74	89.94	89.69	89.91	89.90	89.90	87.07	86.61	87.10	86.48	86.56	86.42
V. campbellii	CCS02	87.15	87.16	87.05	87.06	87.22	89.80	89.79	89.81	90.03	89.84	89.78	89.87	87.31	87.51	87.32	86.29	86.37	86.30
V. campbellii	DS40M4	86.91	86.93	86.80	86.88	86.93	89.50	89.55	89.58	89.61	89.52	89.59	89.61	86.67	86.63	86.76	86.25	86.28	86.30
V. campbellii	HY01	86.87	87.03	86.92	86.85	87.03	89.63	89.65	89.62	89.84	89.59	89.62	89.62	86.78	87.00	86.91	86.19	86.21	86.20
V. campbellii	PEL22A	86.98	86.94	86.84	86.91	86.95	89.63	89.63	89.68	89.76	89.65	89.70	89.69	86.93	86.92	86.95	86.26	86.32	86.35
V. harveyi	NBRC 15634^{T}	86.26	86.25	86.14	86.22	86.25	88.58	88.27	88.42	88.40	88.28	88.26	88.36	86.00	86.10	86.56	85.58	85.64	85.60
V. harveyi	823TEZ1	86.56	86.63	86.43	86.36	86.65	88.96	88.39	88.87	88.87	88.69	88.71	88.85	86.17	86.25	86.80	85.80	85.80	85.83
V. harveyi	AOD131	86.05	86.03	85.93	86.09	86.08	88.31	88.10	88.13	88.08	88.00	87.92	88.12	85.44	85.77	86.16	85.26	85.27	85.28
V. harveyi	CAIM 1792	86.24	86.20	86.18	86.28	86.23	88.56	88.30	88.45	88.42	88.32	88.33	88.42	85.76	85.96	86.42	85.57	85.56	85.58
V. harveyi	VHJR7	86.29	86.33	86.29	86.29	86.25	88.57	88.29	88.40	88.41	88.30	88.29	88.42	86.00	86.17	86.53	85.58	85.60	85.62
V. harveyi	ZJ0603	86.16	86.17	86.11	86.19	86.20	88.42	88.13	88.27	88.27	88.16	88.14	88.31	85.76	86.05	86.37	85.41	85.45	85.37
V. jasicida	LMG 25398^{T}	---	**97.82**	**97.69**	**97.49**	**97.68**	89.20	89.06	89.13	89.24	89.35	89.24	89.29	84.95	84.97	85.60	93.33	93.44	93.35
V. jasicida	090810c	**97.82**	---	**97.83**	**97.46**	**97.71**	89.21	89.07	89.26	89.19	89.31	89.22	89.38	85.05	85.02	85.57	93.38	93.51	93.40
V. jasicida	200612G	**97.76**	**97.91**	---	**97.36**	**97.68**	89.03	89.00	89.18	89.07	89.13	89.16	89.23	84.86	84.94	85.39	93.67	93.79	93.62
V. jasicida	201212A	**97.55**	**97.52**	**97.36**	---	**97.54**	88.97	88.98	89.01	88.93	89.12	89.10	89.08	84.74	84.90	85.34	93.40	93.49	93.31
V. jasicida	MWB 21	**97.66**	**97.63**	**97.54**	**97.50**	---	89.10	88.96	89.14	89.19	89.22	89.27	89.32	85.01	84.92	85.52	93.30	93.42	93.36
V. owensii	LMG 25443^{T}	89.35	89.40	89.18	89.26	89.35	---	**96.54**	**96.44**	**96.43**	**96.69**	**96.32**	**96.74**	86.36	86.42	87.62	87.75	87.83	87.76
V. owensii	051011B	89.08	89.12	89.00	89.04	89.04	**96.54**	---	**96.53**	**96.47**	**96.93**	**96.33**	**96.88**	86.07	86.28	87.43	87.59	87.65	87.70
V. owensii	1DA3	89.13	89.29	89.21	89.09	89.17	**96.39**	**96.53**	---	**96.35**	**96.47**	**96.58**	**96.61**	86.09	86.21	87.36	87.75	87.82	87.74
V. owensii	47666-1	89.29	89.34	89.07	88.99	89.34	**96.38**	**96.45**	**96.45**	---	**96.49**	**96.37**	**96.65**	86.42	86.51	87.80	87.69	87.82	87.73
V. owensii	ATCC 25919	89.33	89.34	89.14	89.17	89.26	**96.63**	**96.88**	**96.41**	**96.49**	---	**96.39**	**96.76**	86.17	86.29	87.50	87.72	87.85	87.70
V. owensii	LMG 25430	89.27	89.31	89.17	89.22	89.30	**96.30**	**96.20**	**96.56**	**96.30**	**96.43**	---	**96.48**	86.14	86.25	87.43	87.96	88.05	87.87
V. owensii	OCN002	89.35	89.47	89.29	89.15	89.44	**96.69**	**96.88**	**96.69**	**96.68**	**96.84**	**96.50**	---	86.41	86.58	87.79	87.91	87.95	87.88
V. rotiferianus	LMG 21460^{T}	85.05	85.10	84.94	84.95	85.13	86.32	86.18	86.18	86.24	86.26	86.15	86.26	---	**97.44**	**96.28**	84.52	84.61	84.58
V. rotiferianus	DAT722	84.99	85.02	84.95	85.01	84.98	86.40	86.36	86.30	86.37	86.43	86.19	86.47	**97.42**	---	**96.63**	84.54	84.53	84.57
V. rotiferianus	Oz08	86.51	86.34	86.05	85.84	86.39	88.35	87.60	88.30	88.42	88.24	88.10	88.33	**96.33**	**96.71**	---	85.32	85.28	85.28
Vibrio sp.	090810a	93.47	93.51	93.72	93.48	93.42	87.71	87.62	87.84	87.69	87.78	87.95	87.91	84.48	84.54	84.99	---	**97.67**	**97.50**
Vibrio sp.	100512A	93.58	93.60	93.80	93.58	93.54	87.77	87.68	87.91	87.81	87.85	87.96	87.96	84.48	84.48	84.88	**97.63**	---	**97.44**
Vibrio sp.	151112A	93.44	93.46	93.65	93.33	93.42	87.64	87.67	87.78	87.64	87.74	87.79	87.86	84.47	84.50	84.89	**97.41**	**97.40**	---

ANI values are presented as percentage. ANI values of 95% or higher are marked in bold.

Supplementary table S2.

Supplementary table S2

Number of identified recombination events. Blue: analysis of interspecies recombination events, orange: analysis recombination events in strains isolated in the Miyazaki prefecture within a short time frame, green: analysis of intraspecies recombination events.

Strains used in the analysis				Number of identified recombination events	Average ANI between strains from different clades
Vibrio campbellii 051011E	*V. campbellii* 200612B	*V. harveyi* NBRC 15634^{T}	*V. harveyi* ZJ0603	6	87.92
V. campbellii 051011E	*V. campbellii* 200612B	*V. harveyi* CAIM 1792	*V. harveyi* VHJR7	9	88.02
V. campbellii 051011E	*V. campbellii* 200612B	*V. jasicida* LMG 25398^{T}	*V. jasicida* 200612G	7	87.15
V. campbellii 051011E	*V. campbellii* 200612B	*V. jasicida* 090810c	*V. jasicida* MWB 21	11	87.21
V. campbellii 051011E	*V. campbellii* 200612B	*V. owensii* ATCC 25919	*V. owensii* 051011B	13	89.74
V. campbellii 051011E	*V. campbellii* 200612B	*V. owensii* 1DA3	*V. owensii* LMG 25443^{T}	10	89.76
V. campbellii 051011E	*V. campbellii* 200612B	*V. rotiferianus* LMG 21460^{T}	*V. rotiferianus* Oz08	31	87.35
V. campbellii 151112C	*V. campbellii* HY01	*V. harveyi* NBRC 15634^{T}	*V. harveyi* ZJ0603	11	87.77
V. campbellii 151112C	*V. campbellii* HY01	*V. harveyi* CAIM 1792	*V. harveyi* VHJR7	6	87.87
V. campbellii 151112C	*V. campbellii* HY01	*V. jasicida* LMG 25398^{T}	*V. jasicida* 200612G	2	87.03
V. campbellii 151112C	*V. campbellii* HY01	*V. jasicida* 090810c	*V. jasicida* MWB 21	9	87.11
V. campbellii 151112C	*V. campbellii* HY01	*V. owensii* ATCC 25919	*V. owensii* 051011B	14	89.68
V. campbellii 151112C	*V. campbellii* HY01	*V. owensii* 1DA3	*V. owensii* LMG 25443^{T}	12	89.71
V. campbellii 151112C	*V. campbellii* HY01	*V. rotiferianus* LMG 21460^{T}	*V. rotiferianus* Oz08	31	87.16
V. campbellii 151112C	*V. campbellii* HY01	*Vibrio* sp. 151112A	*Vibrio* sp. 090810a	7	86.32
V. campbellii DS40M4	*V. campbellii* NBRC 15631^{T}	*V. harveyi* NBRC 15634^{T}	*V. harveyi* ZJ0603	0	87.74
V. campbellii DS40M4	*V. campbellii* NBRC 15631^{T}	*V. harveyi* CAIM 1792	*V. harveyi* VHJR7	0	87.83
V. campbellii DS40M4	*V. campbellii* NBRC 15631^{T}	*V. jasicida* LMG 25398^{T}	*V. jasicida* 200612G	3	86.84
V. campbellii DS40M4	*V. campbellii* NBRC 15631^{T}	*V. jasicida* 090810c	*V. jasicida* MWB 21	2	86.90
V. campbellii DS40M4	*V. campbellii* NBRC 15631^{T}	*V. owensii* ATCC 25919	*V. owensii* 051011B	6	89.46
V. campbellii DS40M4	*V. campbellii* NBRC 15631^{T}	*V. owensii* 1DA3	*V. owensii* LMG 25443^{T}	5	89.52
V. campbellii DS40M4	*V. campbellii* NBRC 15631^{T}	*V. rotiferianus* LMG 21460^{T}	*V. rotiferianus* Oz08	5	86.97
V. campbellii DS40M4	*V. campbellii* NBRC 15631^{T}	*Vibrio* sp. 151112A	*Vibrio* sp. 090810a	0	86.28
V. harveyi NBRC 15634^{T}	*V. harveyi* ZJ0603	*V. jasicida* LMG 25398^{T}	*V. jasicida* 200612G	6	86.16
V. harveyi NBRC 15634^{T}	*V. harveyi* ZJ0603	*V. jasicida* 090810c	*V. jasicida* MWB 21	13	86.20
V. harveyi NBRC 15634^{T}	*V. harveyi* ZJ0603	*V. owensii* ATCC 25919	*V. owensii* 051011B	25	88.18
V. harveyi NBRC 15634^{T}	*V. harveyi* ZJ0603	*V. owensii* 1DA3	*V. owensii* LMG 25443^{T}	31	88.43
V. harveyi NBRC 15634^{T}	*V. harveyi* ZJ0603	*V. rotiferianus* LMG 21460^{T}	*V. rotiferianus* Oz08	21	86.34
V. harveyi NBRC 15634^{T}	*V. harveyi* ZJ0603	*Vibrio* sp. 151112A	*Vibrio* sp. 090810a	4	85.50
V. harveyi CAIM 1792	*V. harveyi* VHJR7	*V. jasicida* LMG 25398^{T}	*V. jasicida* 200612G	9	86.23
V. harveyi CAIM 1792	*V. harveyi* VHJR7	*V. jasicida* 090810c	*V. jasicida* MWB 21	16	86.22
V. harveyi CAIM 1792	*V. harveyi* VHJR7	*V. owensii* ATCC 25919	*V. owensii* 051011B	15	87.76
V. harveyi CAIM 1792	*V. harveyi* VHJR7	*V. owensii* 1DA3	*V. owensii* LMG 25443^{T}	36	88.51
V. harveyi CAIM 1792	*V. harveyi* VHJR7	*V. rotiferianus* LMG 21460^{T}	*V. rotiferianus* Oz08	15	86.38
V. harveyi CAIM 1792	*V. harveyi* VHJR7	*Vibrio* sp. 151112A	*Vibrio* sp. 090810a	2	85.58
V. jasicida LMG 25398^{T}	*V. jasicida* 200612G	*V. owensii* ATCC 25919	*V. owensii* 051011B	29	89.14
V. jasicida LMG 25398^{T}	*V. jasicida* 200612G	*V. owensii* 1DA3	*V. owensii* LMG 25443^{T}	27	89.18
V. jasicida LMG 25398^{T}	*V. jasicida* 200612G	*V. rotiferianus* LMG 21460^{T}	*V. rotiferianus* Oz08	13	85.42
V. jasicida LMG 25398^{T}	*V. jasicida* 200612G	*Vibrio* sp. 151112A	*Vibrio* sp. 090810a	51	93.53
V. jasicida 090810c	*V. jasicida* MWB 21	*V. owensii* ATCC 25919	*V. owensii* 051011B	29	89.17
V. jasicida 090810c	*V. jasicida* MWB 21	*V. owensii* 1DA3	*V. owensii* LMG 25443^{T}	27	89.24
V. jasicida 090810c	*V. jasicida* MWB 21	*V. rotiferianus* LMG 21460^{T}	*V. rotiferianus* Oz08	17	85.51
V. jasicida 090810c	*V. jasicida* MWB 21	*Vibrio* sp. 151112A	*Vibrio* sp. 090810a	63	93.41
V. owensii ATCC 25919	*V. owensii* 051011B	*V. rotiferianus* LMG 21460^{T}	*V. rotiferianus* Oz08	37	86.93
V. owensii ATCC 25919	*V. owensii* 051011B	*Vibrio* sp. 151112A	*Vibrio* sp. 090810a	8	87.69
V. owensii 1DA3	*V. owensii* LMG 25443^{T}	*V. rotiferianus* LMG 21460^{T}	*V. rotiferianus* Oz08	42	87.07
V. owensii 1DA3	*V. owensii* LMG 25443^{T}	*Vibrio* sp. 151112A	*Vibrio* sp. 090810a	12	87.75
V. rotiferianus LMG 21460^{T}	*V. rotiferianus* Oz08	*Vibrio* sp. 151112A	*Vibrio* sp. 090810a	15	84.82
V. campbellii ATCC BAA-1116	*V. campbellii* PEL22A	*V. harveyi* NBRC 15634^{T}	*V. harveyi* ZJ0603	4	87.70
V. campbellii ATCC BAA-1116	*V. campbellii* PEL22A	*V. harveyi* CAIM 1792	*V. harveyi* VHJR7	5	87.86
V. campbellii ATCC BAA-1116	*V. campbellii* PEL22A	*V. jasicida* LMG 25398^{T}	*V. jasicida* 200612G	7	86.99
V. campbellii ATCC BAA-1116	*V. campbellii* PEL22A	*V. jasicida* 090810c	*V. jasicida* MWB 21	8	87.03
V. campbellii ATCC BAA-1116	*V. campbellii* PEL22A	*V. owensii* ATCC 25919	*V. owensii* 051011B	15	89.67
V. campbellii ATCC BAA-1116	*V. campbellii* PEL22A	*V. owensii* 1DA3	*V. owensii* LMG 25443^{T}	15	89.73
V. campbellii ATCC BAA-1116	*V. campbellii* PEL22A	*V. rotiferianus* LMG 21460^{T}	*V. rotiferianus* Oz08	24	87.09
V. campbellii ATCC BAA-1116	*V. campbellii* PEL22A	*Vibrio* sp. 151112A	*Vibrio* sp. 090810a	5	86.34
V. campbellii ATCC BAA-1116	*V. campbellii* DS40M4	*V. harveyi* NBRC 15634^{T}	*V. harveyi* ZJ0603	2	87.73
V. campbellii ATCC BAA-1116	*V. campbellii* DS40M4	*V. harveyi* CAIM 1792	*V. harveyi* VHJR7	3	87.87
V. campbellii ATCC BAA-1116	*V. campbellii* DS40M4	*V. jasicida* LMG 25398^{T}	*V. jasicida* 200612G	6	86.96
V. campbellii ATCC BAA-1116	*V. campbellii* DS40M4	*V. jasicida* 090810c	*V. jasicida* MWB 21	7	87.01
V. campbellii ATCC BAA-1116	*V. campbellii* DS40M4	*V. owensii* ATCC 25919	*V. owensii* 051011B	11	89.63
V. campbellii ATCC BAA-1116	*V. campbellii* DS40M4	*V. owensii* 1DA3	*V. owensii* LMG 25443^{T}	10	89.69
V. campbellii ATCC BAA-1116	*V. campbellii* DS40M4	*V. rotiferianus* LMG 21460^{T}	*V. rotiferianus* Oz08	22	87.10
V. campbellii ATCC BAA-1116	*V. campbellii* DS40M4	*Vibrio* sp. 151112A	*Vibrio* sp. 090810a	3	86.31
V. campbellii 151112C	*V. campbellii* 051011E	*V. jasicida* 090810c	*V. jasicida* 200612G	14	87.16
V. campbellii 151112C	*V. campbellii* 051011E	*Vibrio* sp. 151112A	*Vibrio* sp. 090810a	3	86.37
V. jasicida 090810c	*V. jasicida* 200612G	*Vibrio* sp. 151112A	*Vibrio* sp. 090810a	62	93.55
V. campbellii 051011E	*V. campbellii* 200612B	*V. jasicida* 090810c	*V. jasicida* 200612G	11	87.13
V. campbellii 051011E	*V. campbellii* 200612B	*Vibrio* sp. 151112A	*Vibrio* sp. 090810a	4	86.33
V. campbellii 051011E	*V. campbellii* 200612B	*V. campbellii* DS40M4	*V. campbellii* NBRC 15631^{T}	47	96.27
V. campbellii 151112C	*V. campbellii* HY01	*V. campbellii* DS40M4	*V. campbellii* NBRC 15631^{T}	62	96.05
V. campbellii 051011E	*V. campbellii* 200612B	*V. campbellii* ATCC BAA-1116	*V. campbellii* PEL22A	191	96.53
V. campbellii 151112C	*V. campbellii* HY01	*V. campbellii* ATCC BAA-1116	*V. campbellii* PEL22A	229	96.39
V. campbellii 051011E	*V. campbellii* 200612B	*V. campbellii* ATCC BAA-1116	*V. campbellii* DS40M4	162	96.34
V. campbellii 151112C	*V. campbellii* HY01	*V. campbellii* ATCC BAA-1116	*V. campbellii* DS40M4	203	96.22
V. campbellii 051011E	*V. campbellii* 200612B	*V. campbellii* NBRC 15631^{T}	*V. campbellii* ATCC BAA-1116	154	96.42
V. campbellii 151112C	*V. campbellii* HY01	*V. campbellii* NBRC 15631^{T}	*V. campbellii* ATCC BAA-1116	224	96.21
V. campbellii 051011E	*V. campbellii* 200612B	*V. campbellii* NBRC 15631^{T}	*V. campbellii* PEL22A	100	96.39
V. campbellii 151112C	*V. campbellii* HY01	*V. campbellii* NBRC 15631^{T}	*V. campbellii* PEL22A	138	96.21
V. campbellii 051011E	*V. campbellii* 200612B	*V. campbellii* DS40M4	*V. campbellii* ATCC BAA-1116	163	96.34
V. campbellii 151112C	*V. campbellii* HY01	*V. campbellii* DS40M4	*V. campbellii* ATCC BAA-1116	203	96.22
V. campbellii 051011E	*V. campbellii* 200612B	*V. campbellii* DS40M4	*V. campbellii* PEL22A	119	96.31
V. campbellii 151112C	*V. campbellii* HY01	*V. campbellii* DS40M4	*V. campbellii* PEL22A	138	96.23

Supplementary figure S1.

Phylogenetic resolution of the ‘Harveyi clade’ using parsimonious analysis.

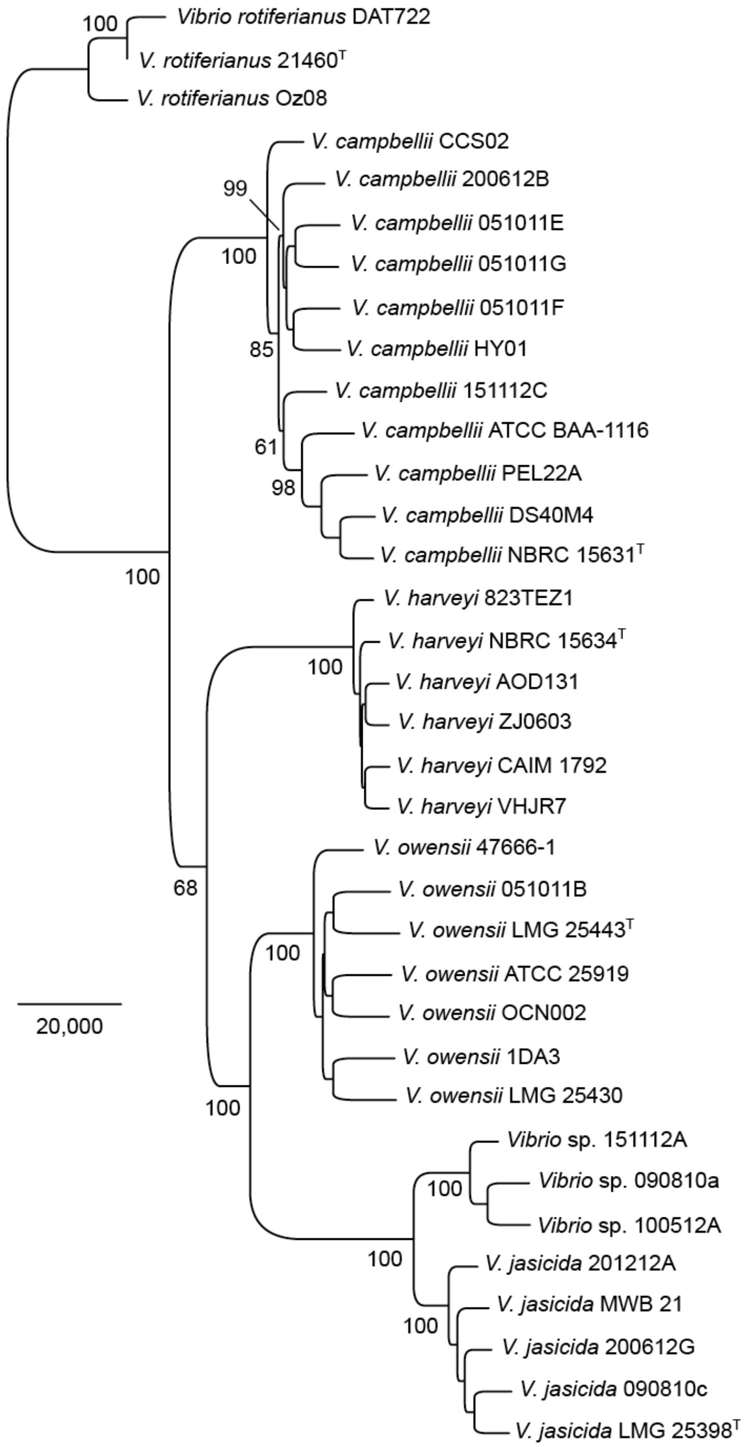

For the analysis an alignment of concatenated sequences of 897 protein coding sequences conserved in the six *Vibrio* species was used. A single most parsimonious tree was found; tree length was equal to 672,871. Jackknife resampling values are reported at the nodes, some omitted for clarity.

Supplementary figure S2.

Relationship between the number of interspecies recombination events and the average nucleotide identity (ANI). Each marker represents the number of recombination events identified between two pairs of strains from different species plotted against average ANI between strains from different species. A linear trend line is shown.

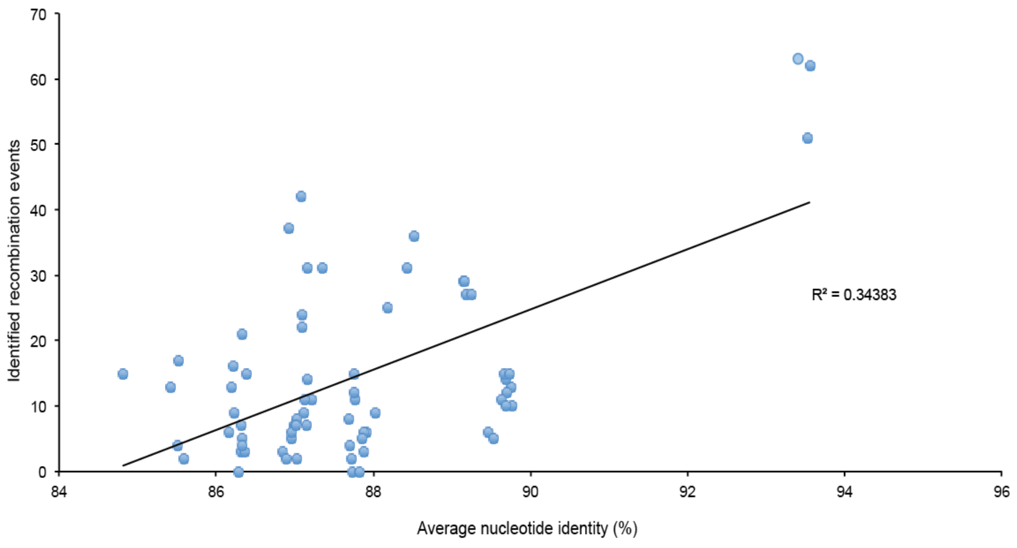

Supplementary figure S3.

Graphic representation of chromosomal location of 895 protein coding genes conserved in the ‘Harveyi clade’ on the map of *V. campbellii* ATCC BAA-1116 chromosomes. Each rectangle represents 500 (or less) protein coding genes organized in the same order as on the ATCC BAA-1116 chromosomes, starting from the origin of replication. 895 conserved sequences are marked red. Blue rectangles indicate sequences showing evidence of recombination between *V. campbellii* strains identified during six analyses of intraspecies recombination events that included strain ATCC BAA-1116.

The analyses (from top to bottom) are:

1. *V. campbellii* 051011E + *V. campbellii* 200612B and *V. campbellii* ATCC BAA-1116 + *V. campbellii* PEL22A
2. *V. campbellii* 151112C + *V. campbellii* HY01 and *V. campbellii* ATCC BAA-1116 + *V. campbellii* PEL22A
3. *V. campbellii* 051011E + *V. campbellii* 200612B and *V. campbellii* ATCC BAA-1116 + V. *campbellii* DS40M4
4. *V. campbellii* 151112C + *V. campbellii* HY01 and *V. campbellii* ATCC BAA-1116 + *V. campbellii* DS40M4
5. *V. campbellii* 051011E + *V. campbellii* 200612B and *V. campbellii* NBRC 15631T + *V. campbellii* ATCC BAA-1116
6. *V. campbellii* 151112C + *V. campbellii* HY01 and *V. campbellii* NBRC 15631T + *V. campbellii* ATCC BAA-1116

Chromosome I

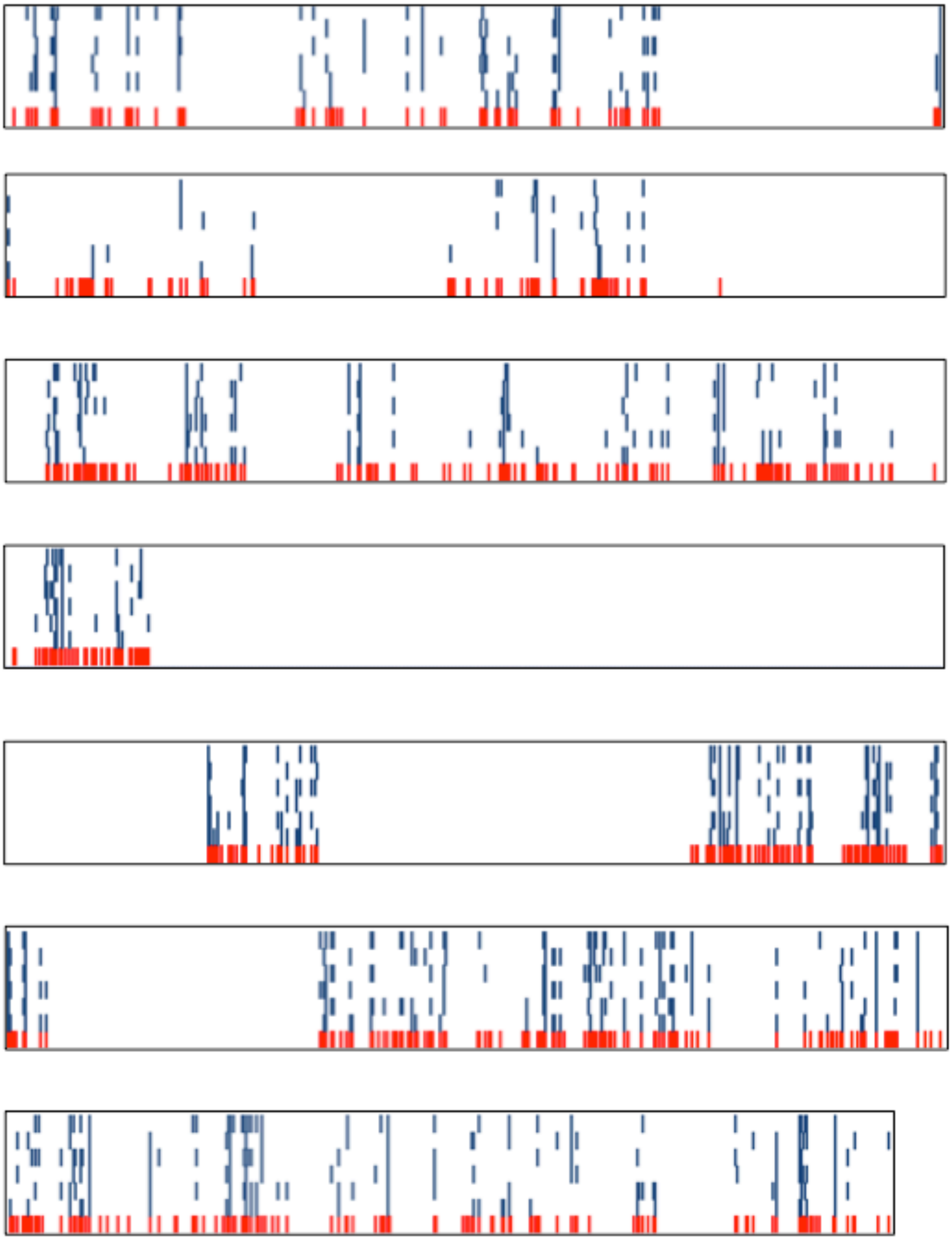

Chromosome II

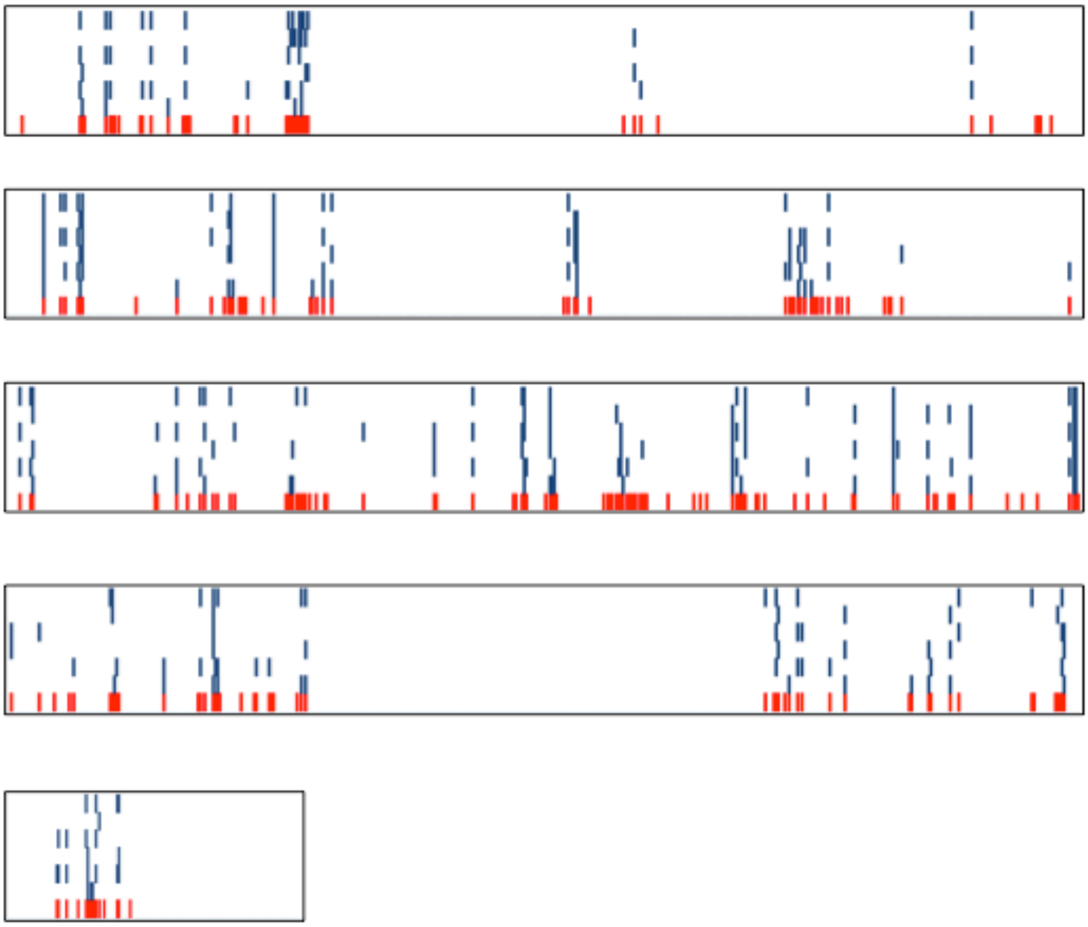

Supplementary figure S4

ClonalFrame estimation of genealogy among 11 *V. campbellii* strains (A), 5 *V. jasicida* and 3 *Vibrio* sp. strains (B), and 3 *Vibrio* sp. and *V. jasicida* 090810c (C). Next to each strain are estimates of recombination (r) and mutations (m) based on ClonalFrame analyses.

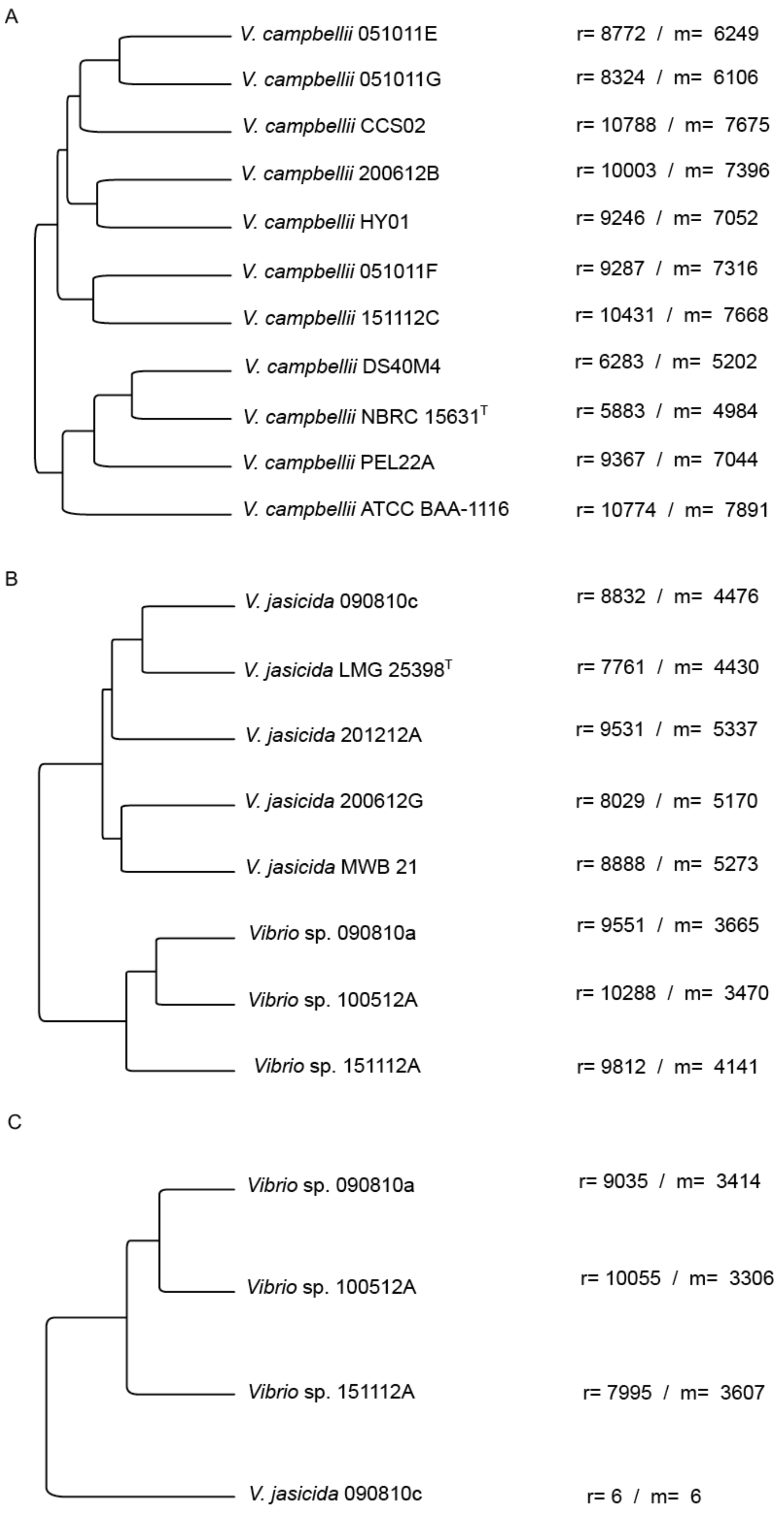
A
V. campbellii 051011E
r= 8772 / m= 6249
V. campbellii 051011G
r= 8324 / m= 6106
V. campbellii CCS02
r= 10788 / m= 7675
V. campbellii 200612B
r= 10003 / m= 7396
V. campbellii HY01
r= 9246 / m= 7052
V. campbellii 051011F
r= 9287 / m= 7316
V. campbellii 151112C
r= 10431 / m= 7668
V. campbellii DS40M4
r= 6283 / m= 5202
V. campbellii NBRC 15631T
r= 5883 / m= 4984
V. campbellii PEL22A
r= 9367 / m= 7044
V. campbellii ATCC BAA-1116
r= 10774 / m= 7891
B
V. jasicida 090810c
r= 8832 / m= 4476
V. jasicida LMG 25398T
r= 7761 / m= 4430
V. jasicida 201212A
r= 9531 / m= 5337
V. jasicida 200612G
r= 8029 / m= 5170
V. jasicida MWB 21
r= 8888 / m= 5273
Vibrio sp. 090810a
r= 9551 / m= 3665
Vibrio sp. 100512A
r= 10288 / m= 3470
Vibrio sp. 151112A
r= 9812 / m= 4141
C
Vibrio sp. 090810a
r= 9035 / m= 3414
Vibrio sp. 100512A
r= 10055 / m= 3306
Vibrio sp. 151112A
r= 7995 / m= 3607
V. jasicida 090810c
r= 6 / m= 6
